# Advances in Targeted Therapy and Immunotherapy for Non-small Cell Lung Cancer Based on Accurate Molecular Typing

**DOI:** 10.3389/fphar.2019.00230

**Published:** 2019-03-12

**Authors:** Jingsi Dong, Bingjie Li, Dan Lin, Qinghua Zhou, Depei Huang

**Affiliations:** ^1^Lung Cancer Center of West China Hospital, Sichuan University, Chengdu, China; ^2^The Medical Department, 3D Medicines Inc., Shanghai, China

**Keywords:** NSCLC, targeted therapy, immunotherapy, EGFR, PD-L1, ALK, ROS1

## Abstract

The essence of precision medicine is to achieve the goal of “individualized treatment” through genotyping of patients and targeted therapy. At present, the pathogenic genes of non-small cell lung cancer (NSCLC) have been studied most thoroughly and targeted therapy based on genotyping has been the most successful. This paper focuses on the precision treatment of NSCLC based on genotyping, comparing gene detection methods and summarize the latest progress of NSCLC immunotherapy.

## Introduction

Fundamentally, cancer is a genetic disease caused by gene variations or epigenetic alterations. However, even the same tumor can be caused by different genetic variations, which is the heterogeneity of the tumor. Even patients with the same pathological type of tumor may respond differently to the treatment because of heterogeneity. Precision medicine is an individualized medical model based on the rapid development of genome sequencing technology and the vigorous rise of biological information and big data science. According to the different molecular types of patients, different treatment regimens are the most reliable methods to improve response and reduce adverse reactions. With the continuous progress of biological detection technology, the cost of human gene molecular typing is becoming lower while the accuracy is getting higher, and individualized treatment is gradually becoming reality.

In recent years, the incidence and mortality of lung cancer have shown a sharp rise in the world. What’s more, lung cancer is the most common cause of cancer death worldwide, with 1.38 million people dying every year, accounting for 18.2% of the total cancer deaths (Jemal et al., 2011). It is also the cancer with the highest morbidity and mortality in China, approximately 781,000 new cases and 626,000 deaths had been reported in 2014 (Chen et al., 2014). Based on the data from the Global cancer statistics 2018, it shows that among the males, incidence rate of the NSCLC is 223.0 per 100,000 and mortality rate is 166.6 per 100,000. Besides, in the female, the incidence rate is 182.6 per 100,000 (Bray et al., 2018). Non-small cell lung cancer (NSCLC) accounts for about 80–85% of lung cancer, and its clinical manifestations are complex and diverse. There are many risks associated with surgical management of advanced NSCLC (Zhang et al., 2017a), drug therapy for advanced NSCLC is safer than surgical treatment (Nie et al., 2012). Patients with the same pathological type of NSCLC may have different responses to the same anticancer drug. At present, the pathogenic genes of NSCLC have been studied most thoroughly and targeted therapy based on genotyping has been the most successful. The purpose of this article is to review the accurate treatment of advanced NSCLC based on genotyping, including targeted therapy, immunotherapy, and comparison of several common detection methods.

## Overview of Targeted Therapy of NSCLC

Chemotherapy was the most important treatment for stage III and IV NSCLC patients until targeted therapy was well developed. The generally acknowledged third-generation new chemotherapy drugs combined with platinum regimen have an overall effect resulted in a significant improvement in survival (HR, 0.79; 95% CI, 0.62–1.00; *p* = 0.05) and a 5 years survival improvement of 11% (67% with chemotherapy vs. 56% with observation) (Nagasaka and Gadgeel, 2018), however, the median survival period is only 8–10 months (Li and Liu, 2018). Meanwhile, chemotherapy drugs cannot differentiate tumor cells and normal cells while working, the treatment related adverse reactions are dramatically strong therefore being feared by patients.

It was not until the emergence of targeted therapy based on molecular typing that the survival period of patients with advanced NSCLC was improved to several years, such as the second generation ALK-TKI alectinib (Alecensa) achieved the PFS of first-line NSCLC patients with ALK fusion up to 34.8 months (Peters et al., 2017), and the adverse reactions were greatly reduced, such as the adverse events of grade 3 or higher was lower with the third generation EGFR-TKI osimertinib (Tagrisso, 23%) than with platinum-pemetrexed (47%) (Mok et al., 2017; Peters et al., 2017).

**FIGURE 1:**
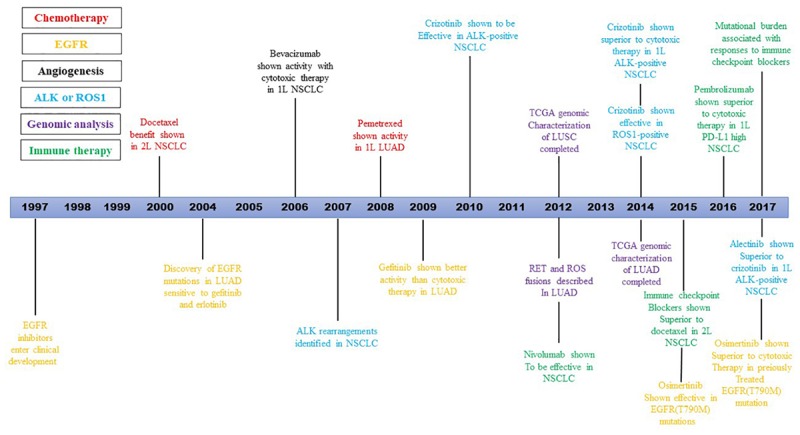
Timeline illustrating the development of targeted therapies and immunotherapies for the treatment of NSCLC over two decades.

The discovery of NSCLC targeted therapy is an event of necessity in contingency. At the end of 2003, researchers from Dana-Farber and Massachusetts general hospital in the United States simultaneously found high remission rates in some NSCLC patients using tyrosine kinase inhibitors (TKIs), and these patients’ high remission rates were confirmed to be the result of EGFR gene mutation (Kris et al., 2003). The first drug, bevacizumab, was approved by the FDA in 2004 for the treatment of advanced colorectal cancer (Herbst et al., 2018). By 2009, the first large randomized controlled study, IPASS, demonstrated that gefitinib significantly prolongs PFS in lung cancer patients with EGFR mutations related to carboplatin-paclitaxel (hazard ratio for progression or death, 0.48; 95% CI, 0.36–0.64; *P* < 0.001) (Mok et al., 2009). An important advance in the management of advanced stage NSCLC occurred in 2015, when the US FDA approved the ICB nivolumab for the treatment of patients whose disease progressed during or after platinum-based therapy, heralding a new era in the management of lung cancer (Herbst et al., 2018). Since then, a series of genes related to the pathogenesis and treatment of NSCLC have been discovered, and a variety of targeted drugs and detection methods have been developed, changing the patterns of advanced NSCLC treatment thoroughly. The latest NSCLC guideline, 2019 v3, published by national comprehensive cancer network (NCCN) suggests that 9 genes related to targeted therapy should be detected, including EGFR, KRAS, HER2, ALK, ROS1, MET, BRAF, RET, and NTRK. Here we use the timeline to show the development of targeted therapies and immunotherapies for the treatment of NSCLC over two decades Figure 1 (Herbst et al., 2018).

### EGFR

As the first therapeutic target discovered, EGFR has been the most thoroughly studied and the most successful. Based on recent studies, EGFR is the most common driving gene in NSCLC in Asia-Pacific and Russian, with an incidence of 49.3% (Han et al., 2017). Mutation types mainly include single nucleotide variation (SNV), insertion, deletion and copy number variation (CNV). The variations were mostly concentrated in exons 18–21, and the responses of exon 19 and 21 to EGFR-tyrosine kinase inhibitor (EGFR-TKI) were generally better than exons 18 and 20. The most common sensitive mutations are the deletion of amino acids at 747–750 of exon 19 (19Del) and the L858R mutation of exon 21, so the use of first-generation EGFR-TKI, namely gefitinib (Iresa), erlotinib (Trockai) and ecotinib (Kemet sodium), can be considered. Afatinib (Giotrif), the second generation of EGFR-TKI, is an irreversible inhibitor with two targets, EGFR and HER2. It is especially applicable for patients with EGFR-TKI resistance caused by HER2 mutation. Afatinib is effective for certain types of rare EGFR mutations and has been approved by FDA for use with rare EGFR mutations: G719X, L861Q, and S768I (Yang et al., 2015).

Drug resistance is almost inevitable after 8–14 months of the first or second-generation EGFR-TKI treatment (Maemondo et al., 2010; Mitsudomi et al., 2010; Tan et al., 2016). The reasons for drug resistance are varied. The mutation of T790M of EGFR exon 20 is the most common cause of drug resistance, accounting for about 50–60% (Kobayashi et al., 2005; Oxnard et al., 2011; Sequist et al., 2011; Yu et al., 2013). In addition, downstream KRAS, BRAF and other activation mutations, HER2 mutation, MET amplification lead to bypass activation, PTEN lost, and transformation to small-cell lung cancer (SCLC), which are also the mechanisms of acquired drug resistance.

If T790M mutation is detected, we can switch to the third-generation EGFR-TKI osimertinib. Studies have shown that after the first or second -generation EGFR-TKI resistance caused by T790M mutation, the survival period of 7.6m can still be obtained by using osimertinib. AURA3 studies in NSCLC patients with EGFR-T790M mutations showed significantly longer PFS with osimertinib compared with permetrexine (Mok et al., 2017). The FLAURA study showed that regardless of whether T790M mutations were detected, the PFS of the first-line treatment group with osimertinib reached 18.9 months, while the median PFS of the first-line standard treatment of the first-generation EGFR-TKI was only 10.2 months, and patients with osimertinib had a high safety (Soria et al., 2018). Thus, first-line use of osimertinib may have a longer FPS than switching to osimertinib after the resistance of first-generation EGFR-TKI.

Taking targeted drug osimertinib as an example, in NSCLC patients with epidermal growth factor receptor (EGFR) T790M mutation, the median duration of progression-free survival (PFS) was significantly longer with osimertinib than with platinum-pemetrexed (10.1 vs. 4.4 months; HR: 0.30; 95% CI, 0.23–0.41; *P* < 0.001); the objective response rate (ORR) were 71% vs. 31% (odds ratio, 5.39; 95% CI, 3.47–8.48; *P* < 0.001) (Mok et al., 2017).

Unfortunately, even if the initial treatment is very effective, patients still have to face drug resistance after months of using osimertinib. The mutation of C797S of EGFR exon 20 is the most common cause of acquired drug-resistance of osimertinib. Geoffrey and his team found out that non-invasive genotyping of cell-free plasma DNA (cfDNA) is a useful biomarker for prediction of outcome from osimertinib (Oxnard et al., 2016). If C797S and T790M are on different chromosomes (trans-configuration), it will still be sensitive to the combination of the first and third-generation EGFR-TKI (Arulananda et al., 2017). On the other hand, if these two mutations are in the same chromosomes (cis-configuration), EGFR-TKI should no longer be used (Goldberg et al., 2018). Interestingly, NSCLC patients with C797S/T790M/19Del or L858R (triple-mutation) are resistance to EGFR-TKIs, but sensitive to ALK-TKI brigatinib (Alunbrig) (Uchibori et al., 2017).

It has been reported that EGFR L718Q, G796D, L844V, et al. mutations are also an acquired drug-resistance site to osimertinib and first-generation EGFR-TKI, but is sensitive to afatinib (Liu et al., 2017; Zheng et al., 2017). Moreover, EGFR is expressed in many normal epithelial tissues and in many human cancers, including those of colon and rectal. For instance, cetuximab also received approval by the FDA for the treatment of head and neck cancer (Altaha and Abraham, 2007).

### ALK

Anaplastic lymphoma kinase (ALK) fusion accounts for 3–5% of NSCLC, which is the second largest mutant gene found after EGFR gene. ALK is most common fused with echinoderm microtubule-associated protein-like 4 (EML4). EML4 is located at P21 of human chromosome 2, while ALK is located at P23. These two genes form a new gene EML4-ALK through inversion fusion. EML4-ALKfusion has more than 21 different forms according to the location of fracture. The sensitivity of different fusion forms to ALK-TKI was also different. The response rate and PFS of patients fused with exon 13 of EML4 gene and exon 20 of ALK gene to crizotinib (Xalkori) were significantly higher than those of other fusion types (Yoshida et al., 2016).

It was once thought that there was exclusion between ALK fusion and EGFR activating mutations (Crystal and Shaw, 2011), which was actually a misunderstanding. Both EGFR and ALK are strong driving genes, as a result, the presence of either of them is sufficient to form tumors, so the presence of them has been rarely observed before. As the data accumulated, some scientists felt obliged to investigate for the patients’ samples did show these two genetic variants coexist (Wen et al., 2016).

PROFILE series of clinical research evidence all confirmed that compared with chemotherapy, both the response rate and disease control time of crizotinib were significantly better (Blackhall et al., 2017). The response rate of crizotinib for ALK fusion positive patients could reach 60∼80%, while that of chemotherapy drugs was only about 30∼40%. Crizotinib is one of the most significant clinical benefits drugs in targeted therapy of lung cancer.

Unfortunately, after initial response to crizotinib, tumors inevitably relapse and end up with drug-resistant symptoms like brain metastasis. In order to overcome crizotinib resistance, alectinib, ceritinib (Zykadia), brigatinib and other second-generation ALK-TKIs were developed, which are more potent and brain-penetrable (Duruisseaux et al., 2017). Surprisingly, all three second-generation ALK inhibitors achieved significant improvements in the first line NSCLC patients with ALK positive. Next generation agents (alectinib and brigatinib) revealed significant improvement in PFS (HR 0.50 [0.43, 0.57; *p* < 0.00001]), ORR (OR 1.57 [1.21, 2.04; *p* = 0.0006]) in comparison to crizotinib and yielded better response intracranially than crizotinib in terms of objective response rate (OR 5.87 [3.49, 9.87; *p* < 0.00001]) and time to CNS progression (HR 0.25 [0.13, 0.46; *p* < 0.0001]) (Khan et al., 2018; Paik and Dhillon, 2018). What’s more, brigatinib received granted accelerated approval by the United States Food and Drug Administration. In ASCEND-4 study (Soria et al., 2017), the mPFS of ceritinib is 16.6 months. In ALTA-1L study (Camidge et al., 2018), the estimated 12 months PFS rate, brigatinib and crizotinib achieved 67 and 43%, respectively. Especially in ALEX study, alectinib was head-to-head compared with crizotinib in first-line advanced ALK-positive NSCLC (Peters et al., 2017). The updated follow-up results was reported in 2018 ASCO conference. The mPFS of first-line alectinib was 34.8 months, compared with 10.9 months for crizotinib, which was amazing 3 times longer. An mPFS of nearly 3 years is enough to allow patients to live for 4–5 years or more, making lung cancer turn into a chronic disease. In addition, significant differences were observed between the two treatment groups regardless of whether the patient’s baseline was associated with brain metastasis. Alectinib was also significantly superior to crizotinib in efficacy duration analysis. Safety data of alectinib is also excellent, and the incidence of serious adverse events remains low even after long-term use. The excellent efficacy and safety of alectinib may be related to its special molecular structure (Kinoshita et al., 2012). The drug structure determines the selectivity and affinity with the target. The higher the selectivity, the higher the safety and efficacy. Alectinib also has excellent blood-brain barrier permeability, which means better treatment and even prevention of brain metastases, and therefore longer PFS. Overall, ceritinib, brigatinib and alectinib have become the first-line treatment scheme recommended in NCCN guidelines for ALK fusion positive patients.

However, very similar to the case of EGFR-TKI, the second-generation ALK-TKIs also face resistance problems. Mutations in the ALK tyrosine kinase domain, such as G1202R, may affect the binding of ALK-TKIs, leading to drug resistance (Gainor et al., 2016). Fortunately, for patients with alectinib resistance, we can also choose the third-generation ALK-TKI lorlatinib (Lorbrena), which is specifically designed for drug resistance mutation sites and has a high blood-brain barrier permeability. By virtue of data already disclosed in the phase I/II studies (Basit et al., 2017; Shaw et al., 2017), lorlatinib has been approved by FDA as a breakthrough drug, and the phase III CROWN study is ongoing. Entrectinib was shown to be well tolerated and active against those gene fusions in solid tumors, including in patients with primary or secondary CNS disease (Drilon et al., 2017).

### ROS1

Human ROS1 gene is a transmembrane tyrosine kinase gene of insulin receptor family, located at q21 of chromosome 6. ROS1 rearrangement represents a new and unique molecules subtype of NSCLC, whose frequency is 1–2%, and is common in young non-smoking female lung adenocarcinoma patients. However, ROS1 fusion occurs in up to 5% of patients with negative EGFR and ALK. Due to the low occurrence probability of ROS1 fusion in lung cancer patients, this molecular subtype was often neglected in the past in clinical practice, and patients with ROS1 fusion were often treated with standard regimens such as chemotherapy for first-line. With the development of gene sequencing technology, ROS1 fusion has been truly demonstrated to clinicians.

ROS1 rearrangement is mainly concentrated in exon 32–36, and at least 9 different fusion types have been found in NSCLC, among which CD74-ROS1 and SLC34A2-ROS1 are common (Jun et al., 2012).

Crizotinib and ceritinib have been shown to be effective in ROS1-positive NSCLC patients. A study published in 2014 showed that in NSCLC patients with ROS1 fusion using crizotinib showed amazing anti-tumor activity, with the objective response rate (ORR) being 72% and median duration of response (mDOR) being 17.6 months (Shaw et al., 2014). But for crizotinib, the first-generation ALK inhibitor, which can cause relapse of CNS metastases can be overcome by newer ROS1 inhibitors (Dong et al., 2016).

Similarly, NSCLC patients with ROS1 fusion who are treated with crizotinib may develop drug resistance, especially CNS metastases. Common drug resistance mutations include G2032R and D2033N of ROS1, which can be overcome by cabozantinib and lorlatinib (Katayama et al., 2015; Zou et al., 2015). A newer inhibitors, entrectinib (RXDX-101), is a ROS1, Pan-TRK, and ALK inhibitor with activity in multiple molecularly defined cancer indications. Entrectinib has extremely high anti-tumor activity against cell lines dependent on the pharmacological targets of this drug *in vitro*, which has shown great promise in phase I/II clinical trials (Ardini et al., 2016). One of the major differences between entrectinib and crizotinib is that crizotinib cannot normally penetrate the brain and CNS, and entrectinib has shown effective CNS activity.

### BRAF

BRAF gene mutation is not only one of the mechanisms of EGFR-TKI resistance, but also an important driver gene and target of targeted therapy. BRAF mutations are found in 1.5–3.5% of NSCLC and cause downstream activation of the MAPK signaling pathway (Leonetti et al., 2018). V600E is the most common mutation. Selective BRAF inhibitors, such as dabrafenib (Tafinlar) and trametinib (Mekinist) has been recommended for the first-line and second-line treatment of advanced NSCLC in the NCCN guidelines.

The ORR of dabrafenib monotherapy for NSCLC patients with BRAF-V600E is 33% (95% CI, 23–45%) (Planchard et al., 2016b). In previously untreated metastatic NSCLC patients with BRAF-V600E, dabrafenib combined with trametinib achieved an ORR of 64% (95% CI, 46–79%), with 2 (6%) patients achieving a CR and 21 (58%) a PR (Planchard et al., 2017). Analogously, in pretreated patients the ORR was 63.2% (95% CI, 49.3–75.6%) (Planchard et al., 2016a).

Vemurafenib (Zelboraf) was approved by the FDA for the treatment of unrespectable and metastatic malignant melanoma with BRAF-V600E mutation in 2011. In the VE-BASKET study, vemurafenib was used for NSCLC patients with BRAF-mutant, the ORR was 42% (95% CI, 20–67%). The median PFS was 7.3 months (95% CI, 3.5–10.8%). The 12 months rate of PFS and OS were 23% (95% CI, 6–46%) and 66% (95% CI, 36–85%), respectively (Hyman et al., 2015).

### MET

c-MET is a kind of transmembrane receptors with independent phosphorylation activity, encoded by mesenchymal epithelial transition (MET) gene (Gherardi et al., 2012). The hepatocyte growth factor (HGF) is the specificity ligand of c-MET (Naldini et al., 1991). MET gene amplification, or c-MET protein overexpression, is one of the causes of resistance in NSCLC patients with the first or second-generation EGFR-TKI, accounting for about 5% of resistances (Engelman et al., 2007; Cappuzzo et al., 2009; Yu et al., 2013). Therefore, some researchers believe that the combination of c-MET inhibitor and EGFR-TKI will be a new idea to overcome drug resistance.

There is also a special mutation form of MET genes: exon 14-skipping (Awad et al., 2016). The Cancer Genome Atlas (TCGA) showed that MET exon 14-skipping were present in about 4% (10/230) of lung adenocarcinoma, leading to partial or complete skipping deletion of MET exon14 at mRNA level (Cancer Genome Atlas Research Network, 2014). The NCCN guidelines recommended crizotinib for patients with c-MET protein overexpression, MET gene amplification, and MET exon 14-skipping mutation in NSCLC patients.

### NTRK

Rearrangements including NTRK1, NTRK2, and NTRK3 are in approximately 2–3% NSCLC patients without other driving genes, such as EGFR, KRAS, EML4-ALK, and ROS1 (Ricciuti et al., 2017).

On November 26, 2018, the US FDA accelerated the approval of larotrectinib (LOXO-101, Vitrakvi) for the treatment of locally advanced or metastatic solid tumors in adults and children with neurotropic tyrosine kinase receptor (NTRK) gene fusion, regardless of the region of cancer occurrence.

It is the first broad-spectrum cancer targeting drug that has been approved by the FDA, which target at NTRK fusion mutations and regardless of cancer type. It have been reported in almost every cancer, and shown to be effective in 17 childhood and adult tumors (Berger et al., 2018).

According to the latest data released at the annual meeting of the European society of oncology (ESMO) in October 2018, larotrectinib was able to achieve 80% objective response rate (ORR) in 55 patients with NTRK fusion cancer that could be measured by RECIST 1.1 criteria. It is noteworthy that larotrectinib showed very consistent results in various cancer types.

Entrectinib was also effective for NTRK fusion. In the studies of ALKA-372-001 and STARTRK-1, the ORR of 5 cases with NTRK fusion was 100%, including 3 cases of brain metastasis (Drilon et al., 2017). Results of phase II study STARTRK-2 have been published on 2018 ESMO congress. The result shows that, regardless of whether brain metastases, 54 NTRK fusion patients (sarcoma 24%, NSCLC 19%, sample secretory breast carcinoma 13%, breast cancer 11%, colorectal cancer 7%, bile duct carcinoma and other gynecological tumor, neuroendocrine carcinoma and salivary gland carcinoma and pancreatic cancer) treated by entrectinib all have gain benefit: ORR 57.4%, mDOR 10.4 months, mPFS 11.2 months, and mOS 20.9 months.

### Others

In recent years, due to the progress of molecular subtyping technology, people have gained a new understanding of the biological mechanism of the occurrence and development of NSCLC. Several specific subtypes of driving genes, such as HER2, RET, KRAS, have been discovered, and corresponding targeted drugs have been developed, thus paving the way for the era of personalized medicine of NSCLC.

The incidence of RET fusion in lung adenocarcinoma is unknown and may be anywhere between 0.4 and 2% (Smit, 2017). The drugs target at RET fusion are much less effective than EGFR-TKI or ALK-TKI, and combination therapies may be the key to improving response in the future.

Human epidermal growth factor receptor 2 (HER2) is expressed in many cancers, including NSCLC. HER2 amplification has been reported to occur in up to 13–22.8% of NSCLC (Yu et al., 2013; Ko et al., 2018). Afatinib could be a useful therapeutic agent as HER2-targeted therapy for patients with NSCLC harboring HER2 alterations (Torigoe et al., 2018).

KRAS is a driving gene with very high mutation frequency in NSCLC and colorectal cancer, accounting for 3% in western population and 6% in Asian population (Domagala et al., 2012). KRAS has a high mutation frequency in many other tumor species. However, unfortunately, there are currently no targeted drugs specifically targeting KRAS mutations approved in list. KRAS mutation may affect the efficacy of EGFR-TKI, but increase the efficacy of immunotherapy.

## Overview of Detection Method

With the continuous progress of biological detection technology, the acquisition of human gene molecular typing is becoming more convenient and easier, and accurate treatment is gradually becoming the reality.

According to the different detection objects, there are many detection methods: ARMS, NGS, ddPCR, and FISH are targeted at DNA; RNA was targeted by RNA-Seq and RT-PCR, and IHC for protein. If the amount of tumor tissue is too small or unable to obtain tissue, we can supplement with liquid biopsy through blood, hydrothorax or ascites, pericardial effusion, cerebrospinal fluid and other specimens (Zhang et al., 2017b).

### ARMS

The amplification refractory mutation system (ARMS) based on fluorescence quantitative PCR technology has become the mainstream technology of gene mutation detection due to its simple operation, high specificity, high sensitivity and good repeatability, which is suitable for hospital laboratory to carry out test by itself. ARMS can be used to detect gene mutations in tumor tissues and peripheral blood, but the prerequisite is to specify the specific site to be detected in advance and design the corresponding PCR primer. Therefore, ARMS is only applicable to the detection of known mutation sites and cannot be used for the detection of unknown sites.

### NGS

High-throughput sequencing, or next-generation sequencing (NGS), because of its low cost, large throughput, high accuracy and rich information content, has become a very important role in the study of genome, transcriptome and epigenetics. NGS mainly includes the whole genome sequencing (WGS), whole exome sequencing (WES), as well as the targeted region sequencing (TRS) or cancer gene panel (CGP). Recently, NGS is used to sequence circulating tumor DNA (ctDNA), as a kind of liquid biopsy. ctDNA is derived from necrotic tumor cells, apoptotic tumor cells, circulating tumor cells and exosomes produced by tumor cells. Although ctDNA samples are relatively simple to obtain, they are extremely low in content, accounting for 0.01∼1.00% of cell free DNA (cfDNA) in plasma. It was not until the emergence of NGS technology that the difficulty of detecting mutations from extremely low abundance samples was solved.

The types of mutations detected by NGS mainly include SNV, insertion, deletion, CNV, etc., including known and unknown mutation forms, which can provide much more information than ARMS. However, in terms of laboratory hardware setting and personnel qualification, it has higher requirements than conventional PCR laboratories. Therefore, it cannot be widely used in more hospitals at present, and third-party testing institutions are more likely to provide testing services.

### FISH

Fluorescence *in situ* hybridization (FISH), can be used for the detection of CNV and gene fusion frequently. The number and location of the corresponding genes can be clearly displayed under the microscope by fluorescent probe labeling, and the CNV and fusion can be determined by this method. FISH is the gold standard for the detection of gene fusion. Taking ALK fusion as an example: two probes, red and green, are designed to mark the two ends of ALK gene, respectively, once ALK gene is broken and rearranged or inverted, red and green signals will be separated under the microscope; while those that are not broken will show yellow fluorescence signals. FISH can detect whether a gene has been rearranged or reversed, but it cannot determine where it has been broken or fused with which gene. NGS detects gene fusion to identify the site of rupture and to identify which genes are fused. However, about 10% of fusion fracture sites were located in introns or not contained by probes, so NGS could not detect them. Therefore, NGS combined with FISH will help to determine the final result.

### ddPCR

Droplet digital PCR (ddPCR), as the third generation of PCR technique usually used in ctDNA liquid biopsy, gain a great of attention in the field of clinical diagnosis because of its high sensitivity. By using the micro-PCR amplification system, absolute quantification can be achieved, which overcomes the difficulty of ctDNA not easy to be amplified due to its low content. Same as ARMS, ddPCR can only detect known sites, but it is fast and sensitive, with almost no detection threshold. ddPCR combined with NGS cannot only quickly check whether there are common mutations, but also examine whether there are rare mutations in a wide range.

Due to its high sensitivity, relatively non-invasive and absolute quantification, ddPCR has been used for dynamic monitoring of mutant copy number, which can be used for real-time monitoring of the relationship between tumor progression, drug resistance and gene evolution.

### IHC

Genes ultimately function through proteins, so detection of proteins is of more concern. Immunohistochemistry (IHC) is the detection of protein level, which can detect protein deficiency, overexpression, fusion and so on. Surgical tissue, fine needle aspiration biopsy tissue, pleural or ascites centrifugal precipitates can all be used for the detection of immunohistochemistry.

The test results of DNA level and protein level sometimes do not match. Amplification of a gene usually implies overexpression of the corresponding protein, such as androgen receptor (AR). On the contrary, when the AR protein is overexpressed, but the AR gene is not necessarily amplified, because there are many regulatory mechanisms from DNA to protein. Similarly, if the promoter is methylated, then even if the DNA sequencing is normal, the corresponding protein may be missing. There is also a weak relationship between some genes and protein expression, such as CD274 gene and PD-L1 protein, and there is almost no relationship between the expression level of PD-L1 and the copy number of CD274.

## Overview of Immune Checkpoint Inhibitors

The cause of the tumor is related to immune system oversight. All malignancies originate from genetic mutations, including germline and somatic mutations. According to the genetic central dogma, the changes in DNA ultimately cause changes in proteins. The altered proteins (tumor proteins) are immunogenic (neoantigens). A large number of neoantigens, caused by genetic and epigenetic changes, can induce an immune response, but tumors can induce tolerance between tumors and specific T cells by up-regulating ligands of inhibit receptors, ultimately resisting immune attacks (Mellman et al., 2011). According to the Cancer immunoediting theory first proposed by Dunn et al. (2002), the immune response has three stages of elimination, equilibrium and escape, and it is regulated by various stimulant and inhibitory factors (Dunn et al., 2002). Tumor immunotherapy is to activate the body’s auto defense mechanism or give exogenous substances to regulate the immune response to tumor, and stimulate the immune cells to identify, inhibit and kill tumor cells (Dong et al., 2018). The immune escape mechanism of tumors plays an important role in the occurrence and development of tumors (Siegel et al., 2017).

Currently, inhibitors of two checkpoints, cytotoxic T lymphocyte antigen – 4, (CTLA-4), programmed cell death protein – 1 (PD-1)/programmed cell death ligand 1 (PD-L1), have been approved to be listed. PD-1 inhibitors include nivolumab, pembrolizumab, and PD-L1 inhibitors include atezolizumab, durvalumab, avelumab, etc.

Immunotherapy often produces some magical results and has aroused great interest among oncologists worldwide. However, not every patient can achieve this effect, and some patients may even progress faster after receiving immunotherapy. MDM2/4 amplification, chromosome 11q13 (CCND1, FGF3, FGF4, and FGF19) amplification and EGFR amplification may be associated with hyperprogression in immunotherapy (Kato et al., 2017). Molecular typing is also needed to determine which patients are suitable for use, and biomarkers are used to screen out which patients can use immunotherapy.

### PD-L1

PD-1 is expressed in T cells, natural killer cells (NK), monocytes and B cells. PD-L1 is mainly expressed on the surface of tumor cells and in the tumor microenvironment. When PD-1 binds to PD-L1, T cells are inhibited. PD-L1 on the tumor cell membrane can be abnormally up-regulated and inhibit the activation of T lymphocytes, leading to tumor immune escape (Haanen and Robert, 2015). The PD-L1 expression level is the first biomarker approved by FDA. Although there were some differences reported in the results of clinical trials in which different PD-1/PD-L1 inhibitors were involved, the benefit of immunotherapy was significantly higher in those with high expression of PD-L1 than in those with low expression (Dong et al., 2018).

IHC is the gold standard for detecting the expression rate of PD-L1 protein. There are two platforms (Dako, Ventana) and four testing kits (28-8, 22C3, SP263, SP142) used for PD-L1 detecting.

### TMB

Tumor mutation burden (TMB) is also able to identify candidate patients who will benefit from immunotherapy. TMB is defined as the number of non-synonymous somatic mutations per million bases, excluding germline mutations. Therefore, TMB describes the stability of the genome. The higher the TMB is, the more mutations will be, as a result there will be more new antigens, more immunogenicity, and better effect of immunotherapy.

NGS is the only method for TMB detection. TMB measured by WES is the gold standard, but it is costly, time-consuming and inconvenient for clinical application. The TMB measured by TRS (CGP) avoids these problems successfully and has been widely carried out in clinical practice. The question then becomes how to define “TMB-H.” When different regions were selected for sequencing, the TMB value obtained was inconsistent and an accepted cutoff value could not be determined. A smart solution is to compare the ordering of the absolute value of CGP-TMB instead of comparing the absolute value directly. The CGP-TMB values of the same tumor species detected by the same detection institution can be sorted, with the highest 25% defined as “high,” the middle 50% defined as “medium,” and the lowest 25% defined as “low,” which can also be divided into three equal parts. Blood-based TMB (bTMB) has also been shown to be useful in screening people for immunotherapy (Gandara et al., 2018).

In October 2018, based on the results of Checkmate-227 (Hellmann et al., 2018) and Checkmate-026 (Carbone et al., 2017), the NCCN issued 2019 v1.0 guidelines for NSCLC, recommending TMB for the first time to identify lung cancer patients who are suitable for the combination of “nivolumab + ipilimumab” and “nivolumab.” But it also points out that there is no consensus on how to measure TMB.

### MMR and MSI

Mismatch-repair (MMR) deficiency and microsatellite-instability (MSI) can predict the therapeutic effect of tumor immunotherapy (Le et al., 2015). MSI is major caused by MMR- deficient (dMMR), and we detect MSI to determine whether the function of MMR is proficient (pMMR). Therefore, like TMB, MSI, and MMR are indicators of genomic stability. dMMR enables tumors to synthesize new antigens with potential immunogenicity, thus causing an immune response (Colle et al., 2017).

The gold standard for MMR detection is IHC to detect whether four MMR proteins (MLH1, MSH2, MSH6, PMS2) are expressed. There are two possible reasons for the non-expression of MMR protein. 1. MMR gene variation; 2. Methylation of MMR gene promoter. The first cause can be also detected by NGS, but the second can only be confirmed by detecting promoter methylation (McCarthy et al., 2018).

MSI can be measured by PCR or NGS. PCR-MSI is easy to operate, and has been carried out in many hospitals, but only five sites have been detected so far. Compared with PCR, NGS detection has a wider range and can avoid detection omission effectively (Hempelmann et al., 2018). The sensitivity and specificity of CGP-MSI based on NGS can reach 93.1–96.6% and 97.2–100%, respectively (Salipante et al., 2014; Hempelmann et al., 2018). In addition to improved sensitivity, NGS-MSI testing offers several advantages over PCR-MSI methods. (1) They do not require matched non-tumor tissue; (2) Interpretation is streamlined and semiautomated (Hempelmann et al., 2018).

However, dMMR or MSI is a low-probability event in NSCLC.

## Summary

Tumor-targeted therapy is to determine the treatment method for specific driving gene mutations by detecting whether there are gene mutations or gene spectrum changes in tumors that lead to tumor growth. In order to determine whether patients can use targeted therapeutic drugs, genetic molecular typing is needed first.

In the selection of gene detection technology, it is necessary to select appropriate detection technology according to its corresponding target to ensure the accuracy and reliability of detection results.

NSCLC has the most thorough research and the most sufficient evidence in driving gene molecular typing. Accurate treatment of NSCLC patients can only be achieved by using the technology and accurate detection. We hope that in the future, more detection technologies, more driving genes and more effective targeted drugs will emerge, so that NSCLC will become a controllable chronic disease.

## Author Contributions

QZ put forward the content of the paper. JD wrote the manuscript. BL, DL, and DH literature and clinical data were reviewed.

## Conflict of Interest Statement

DH was employed by company 3D Medicines Inc. The remaining authors declare that the research was conducted in the absence of any commercial or financial relationships that could be construed as a potential conflict of interest.
